# Correspondence

**Published:** 1867-06

**Authors:** 


### ARTICLE VII.

Messrs. Editors:—

Happening to ask an esteemed friend and professional brother from the state—I beg pardon, the territory—of Georgia, what he thought of the action of the Pennsylvania College, I was made to feel very much like a reconnoitering party who had come unexpectedly upon a masked battery.

As soon as I had recovered somewhat from the suddenness of the discharge, and found that no bones were broken, I ventured to ask my friend to put a little of his thunder and brimstone on paper. I am afraid his pen was a steel one, and not a goose quill, and his ink had in it rather more of the gall of bitterness, than the genuine Aleppo galls, which the receipt calls for. But the Angel of Mercy, who writes with the pen of justice dipped in the indellible ink of truth, has written so very little of late for our papers and magazines, (having been severely paralyzed six years ago,) that really it would be hypercritical in an editor to object to an article because it smells somewhat of sulphur.

"A college has SECEDED! Had it been the Baltimore College, we should not have wondered, for that rebel city has never been loyal except by compulsion, under force of the argument of bayonets. But it is the Pennsylvania College; in a state overflowing with loyalty and patriotism; a state which has contributed more than perhaps any other towards crushing out a wicked rebellion: the only loyal state whose sacred soil was laid waste by the sacrilegious southron. Alas! that the bitter teachings of four years of civil war should so soon be forgotten, that in the very hot-bed of patriotism, this noxious weed of secession should dare to spring up. Surely the whirlwind of war must have scattered the seeds of evil growth far and wide.

"The Constitution and the Union" of the United Colleges is in danger. A rebellious minority rejects our wise amendments to that constitution, and proposes a scheme, which practically "nullifies" the great principles of union. How shall we meet the issue, and maintain intact the grand truths, established by our late glorious struggle—that a state (a college) has no rights; that a minority has no rights; that the voice of the people (i. e. the majority) is the voice of God? What measures shall we adopt, to re-establish a "Republican form of government" in the profession, and to show that our system is the "best the world ever saw."

"O for a Sumner to speak in classic strains of the "barbarism" of these rebels: a Butler to enforce *taking* plans for their subjugation; a Stevens to elaborate some scheme of confiscation, which, like his last, shall so exquisitely temper justice with mercy. Let our watchwords be "Might makes right." "The end justifies the means." "The people can do no wrong;" and for our Marsellaise let us sing that "He who has the power may take,

And he may keep who can:"

for in these grand sentiments lies the death knell of secession, and of all attempts of any minority to control or dictate to, the omnipotent and infallible majority.

"We will take a lesson from Judge Advocates, and accept insufficient or manufactured evidence in condemnation of rebels; for "justice to traitors is treason to loyalty." On the testimony of a student, who was refused admission, and of another who was rejected on examination, we are prepared to prove that the Professors in this "seceded college" are unfit to teach dentistry. Our sentence would be that their Diplomas be revoked; that all loyal men having their photographs "turn them to the wall" and that their professional life meet the fate of Mrs. Surratt.

"We will take a lesson from pious New England. For if "if rebellion is as the sin of witchcraft," and the witch could be purified only by "fire and faggot," what severity of punishment is too great for the rebel.

"We will take yet another lesson from the late war, and close their ports of entry. They shall have no students; or, if any run the blockade, they shall not be recognized as members of a loyal profession, but shall suffer the penalty of perpetual disfranchisement. We will declare "contraband of war" all the "medicines and necessaries of [professional] life" and then when we have rescued some wretched victim of their starving Andersonville-like system of teaching, we will distribute, broadcast throughout the profession, mental photographs of this unanswerable proof of their depraved and inhuman practices."

At this point, I ventured to arrest the flow of patriotic enthusiasm, in my "reconstructed" rebel friend. I could easily understand how, in his strong desire to make up, by excess of loyalty, for past defection, he might go beyond the mark: and could excuse his indignation at seeing things allowed at the North, which were so mercilessly punished at the South. But I could see plainly, in the very language used, that he was confounding ideas political with things intellectual and educational; and that, if his arguments had any force, it could only be by bringing our Dental Colleges down to the sumner—stevens—butler level. This could not be permitted.

The unanswerable logic of conquest may prove that states and minorities have no longer any "rights:" but in the kingdom of science and art the appeals of reason and justice are not as yet made in vain. The Pennsylvania school has the same right to secede that South Carolina had under the old constitution; but the college had perhaps less excuse or provocation than the state found in view of grievances, past, present or prospective. It may be the Northern college will find secession as unwise a remedy as did the unfortunate Southern state.

I propose elsewhere to consider at some length this action of the Pennsylvania school, and shall not speak further of it here. I only make these remarks least I should be considered as endorsing fully the measures proposed by my rather "too loyal" friend. I suggest their publication, because it is well to look at so important a subject from all possible positions. Perhaps some rampant loyalist, late convert to conservatism, might present the subject from another and quite different point of view.

Yours truly, P. H. A.

---

